# No influence of eye gaze on emotional face processing in the absence of conscious awareness

**DOI:** 10.1038/s41598-019-52728-y

**Published:** 2019-11-07

**Authors:** Nathan Caruana, Christine Inkley, Marwa El Zein, Kiley Seymour

**Affiliations:** 1 Perception in Action Research Centre, Macquarie University, Sydney, Australia; 2 Department of Cognitive Science, Macquarie University, Sydney, Australia; 3 Institute of Cognitive Neuroscience, University College London, London, United Kingdom; 4 School of Psychology, Western Sydney University, Sydney, Australia; 5 The MARCS Institute for Brain, Behaviour and Development, Western Sydney University, Sydney, Australia

**Keywords:** Human behaviour, Object vision

## Abstract

The human brain has evolved specialised mechanisms to enable the rapid detection of threat cues, including emotional face expressions (e.g., fear and anger). However, contextual cues – such as gaze direction – influence the ability to recognise emotional expressions. For instance, anger paired with direct gaze, and fear paired with averted gaze are more accurately recognised compared to alternate *conjunctions* of these features. It is argued that this is because gaze direction conveys the relevance and locus of the threat to the observer. Here, we used continuous flash suppression (CFS) to assess whether the modulatory effect of gaze direction on emotional face processing occurs outside of conscious awareness. Previous research using CFS has demonstrated that fearful facial expressions are prioritised by the visual system and gain privileged access to awareness over other expressed emotions. We hypothesised that if the modulatory effects of gaze on emotional face processing occur also at this level, then the gaze-emotion *conjunctions* signalling self-relevant threat will reach awareness faster than those that do not. We report that fearful faces gain privileged access to awareness over angry faces, but that gaze direction does not modulate this effect. Thus, our findings suggest that previously reported effects of gaze direction on emotional face processing are likely to occur once the face is detected, where the self-relevance and locus of the threat can be consciously appraised.

## Introduction

Faces provide a rich source of social information (e.g., identity, gender, age, mental perspectives and emotional states), the processing of which is supported by specialised brain regions^1–5^. The accurate perception of social information in faces enables us to effectively understand the perspectives of conspecifics. Emotional expression is a particularly important source of information as it provides rapid insights into the internal states of others and guides how we approach, interact with, or avoid our conspecifics. Thus, accurately evaluating and responding to such cues is critical for maintaining social relationships, but arguably also serves an adaptive function for survival^6–8^.

Emotional expressions conveyed by others, if accurately perceived and categorised, can provide important warning signals of threat and danger to the observer. But while perception of emotional expressions depends primarily on facial configuration, it is also influenced by other face-bound contextual cues. For instance, gaze direction is an important contextual cue known to influence judgments of emotional expression, particularly those that signal threat to the observer (i.e., anger and fear). This is arguably because gaze direction contextualises the relevance and locus of the threat to the observer^9–11^. Specifically, angry faces with direct gaze signal threat directed at the observer, by a conspecific. In contrast, fearful faces with averted gaze indicate an external source of threat in the immediate environment (e.g., a predator)^10^. Indeed, these gaze direction and emotion combinations have been found to result in the subjective perception of the expressed emotion as being more intense than alternate combinations^9,10^. Further, fearful faces have been found to be more frequently detected during an attentional blink paradigm when they display averted rather than direct gaze, whereas angry faces are more frequently detected when they display direct gaze^12^. It has also been shown that certain gaze direction and emotion conjunctions are more accurately and readily recognised than others^13–17^. As such, it has thus been suggested that gaze direction acts to provide additional contextual cues that boost the perceptual salience and valence of the emotional threat signal – because the locus of threat conveyed is of greater relevance to the observer (hereafter referred to as “Threat+” faces) – than that signalled by alternate gaze and emotion conjunctions (i.e., “Threat−” faces)^18^.

Consistent with findings of a heightened perceptual sensitivity to Threat+ faces (anger paired with direct gaze, fear paired with averted gaze) than Threat− faces (anger paired with averted gaze, fear paired with direct gaze) is the idea that evolutionary pressure has led to the hardwiring of specialized brain mechanisms that prioritize the processing and response to threatening stimuli^6^. The ‘low-road’ hypothesis theorises that the rapid responding to threat-related visual cues is supported by a specialized subcortical pathway from the retina to the amygdala, which bypasses the visual cortex and operates outside of visual awareness^19–25^. Ancient brain structures like the superior colliculus and pulvinar are thought to operate in the absence of conscious awareness to rapidly orient attention to the threatening stimulus and facilitate a response^26^. Although neuroanatomical support for this pathway has been limited^27^, recent research shows evidence for an afferent subcortical pulvinar connection to the amygdala that facilitates fear recognition^28^. There is also consistent evidence to suggest that threat is processed outside of visual awareness^29,30^. Continuous flash suppression (CFS) is a technique that suppresses stimuli from visual awareness and is thought to target early subcortical visual processing^31–34^. Experiments using this technique consistently show that faces with fearful expressions gain privileged access to visual awareness over faces with neutral or happy expressions^35–44^. Further, fMRI studies using CFS have also shown that amygdala responses elicited by fearful faces – that are suppressed from awareness – are similar to those elicited by consciously perceived fearful faces^36,45^. These findings support suggestions of a ‘low road’ pathway which operates without awareness to prioritize the detection of threatening stimuli.

Although there is evidence for the processing of fearful faces outside of visual awareness, it is currently unclear whether the modulatory effects of gaze on emotion perception occur at the same preconscious stages of processing. For example, would a fearful face with direct gaze (i.e., a Threat− face) gain access to awareness earlier than an angry face with direct gaze (i.e., a Threat + face), despite the angry face presenting a more relevant threat signal to the observer? Some behavioural evidence from studies using the Garner Interference task show that when expressed emotions are unambiguous, the emotion is encoded before gaze interferes^15^, suggesting that the effect of gaze may occur at later stages of cognitive processing. However, evidence from electroencephalography (EEG) studies suggest that neural responses to human gaze direction and emotional expression interact as early as 200 milliseconds after stimulus presentation^11,17,46–48^. In recent studies, researchers have created stimuli with different levels of perceived threat using different combinations of identical gaze directions (direct and averted) and expressed emotions (angry and fear)^18,48^. By matching the gaze directions and emotion expression across conditions, they were able to identify brain responses caused by the *conjunction* of gaze direction and emotion, and thus the perceptual integration of these cues, while controlling out activation produced by either gaze direction or emotion expression alone. They found support for perceptual integration in ventral face-selective and dorsal motor cortices 170 ms after the stimulus presentation, as evidenced by enhanced neural responses to Threat + conjunctions compared to Threat− conjunctions. This suggests that the detection and behavioural response to self-relevant threat signals are supported by fast-acting neural circuits. However, no study has yet assessed whether the fast integration of gaze and emotion cues occurs outside of awareness leading to the faster detection of Threat + stimuli.

**Figure 1:**
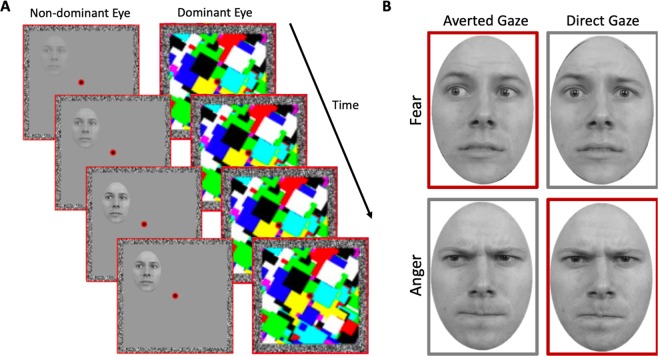
(**A**) A schematic example of a b-CFS trial using fearful face stimuli with averted gaze. Participants were presented with masks flashing at 10 Hz to their dominant eye, whilst an image of a face was presented to their non-dominant eye. The mask presented to the dominant eye resulted in the temporary suppression of the face stimulus from conscious awareness. Participants were required to press a button as quickly and accurately as possible to indicate whether the face stimulus appeared on the left or right side of the frame. (**B**) An example stimulus set from one of the 24 face identities presented using the b-CFS paradigm. Red and grey borders indicate Threat+ and Threat− faces, respectively. Importantly, the two Threat +and two Threat− conditions contained the same two gaze directions and emotional expressions but differed exclusively in their unique feature *conjunctions*. Thus, any measured difference in suppression times for Threat+ and Threat− stimuli cannot be explained by the separate features alone.

In the current study, we aimed to use the breaking-CFS (b-CFS) paradigm (Fig. 1) to test whether the modulatory effects of gaze direction on emotional face processing occur outside of awareness. We hypothesised that if gaze direction and emotion expression is integrated at the earliest preconscious stages of face processing – and acts to boost the early detection of self-relevant threat signals – then Threat+ faces should gain access to awareness faster (i.e., exhibit shorter suppression times) than Threat− faces.

## Results

**Figure 2:**
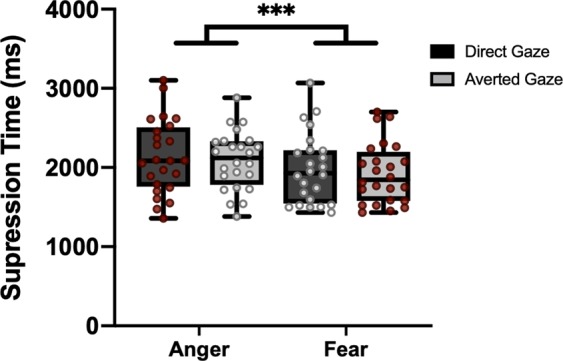
Box plots and individual data points summarising the mean suppression time data by stimulus condition (see Method). Data points with red and white fill indicate responses to Threat+ and Threat− faces, respectively. ***p < 0.001.

Using a b-CFS paradigm, we assessed whether the integration of emotional expression (fear versus anger) and gaze direction (direct versus averted) occurs at early stages of face processing, leading to the prioritised awareness of emotion and gaze *conjunctions* that signal self-relevant threat (Fig. 1). Mean suppression times for each stimulus condition are summarised in Fig. 2. A two-way repeated measures ANOVA with factors: emotion (angry, fear) and gaze direction (direct, averted) revealed a main effect of emotion, with participants being significantly faster to detect fearful faces than angry faces (*F*(23) = 21.89, *p* < 0.001, *η²* = 0.488; mean difference = 158.77 ms, *SD* = 168.88). Our analyses did not reveal evidence for a significant main effect of gaze direction (*F*(23) = 2.09, *p* = 0.162, *η²* = 0.083). Moreover, we did not detect the hypothesised interaction between emotion and gaze direction on suppression times (*F*(23) = 0.241, *p* = 0.628, *η*² = 0.010). This suggests that Threat+ and Threat− conjunctions of expressed emotion and gaze direction did not differentially affect suppression times.

To confirm that there were no differences in suppression times for Threat+ and Threat− face conjunctions, we averaged suppression times across both Threat+ (i.e., anger-direct, fear-averted) and Threat− (i.e., anger-averted, fear-direct) face conditions and compared them using a paired t-test. This analysis revealed no evidence for a significant effect of self-relevant gaze-emotion conjunctions on suppression times (*t*(23) = 0.491, *p* = 0.628, *d* = 0.100).

## Discussion

Using a b-CFS paradigm, we assessed whether the integration of emotional expression and gaze direction occurs at early stages of face processing, leading to the prioritised awareness of emotion and gaze *conjunctions* that signal self-relevant threat (i.e., Threat+). Consistent with previous research^38,41,42,44,49^, our study found that faces with fearful expressions gain privileged access to conscious awareness over angry expressions. However, we found no evidence that gaze direction modulates this effect. These findings suggest that, whilst the effects of gaze direction on emotion processing may act to emphasise the locus and self-relevance of threat^14,17,18,46–48,50,51^, they are likely to manifest after the face is detected and is assigned conscious appraisal.

In a recent study using a similar set of face stimuli to those used here, participants were required to discriminate between fear and anger^18^. When participants were asked to consciously evaluate the face, gaze direction was shown to boost emotion categorization performance. This suggests that the modulatory effects of gaze direction may act to lower *discrimination* thresholds and sharpen the discriminability of threat-signalling emotions, rather than lower the threshold for *detecting* the face. This would be particularly advantageous under conditions of uncertainty, where the face is detected but is poor in signal quality (e.g., in the dark)^52^. In such cases, information about gaze direction would be critical for disambiguating whether the threat is relevant to the observer and thus requiring additional neural and attentional resources. In support of this idea, the effects of gaze^15^ on emotion discrimination are strongest when the face morphology weakly exhibits the expressed emotion^17,53^, compared to when the expressed emotion is more intensely conveyed^18,52^.

Our findings suggest the modulatory effects of gaze direction on emotional face processing requires conscious awareness. This is consistent with previous evidence from speeded emotion categorization tasks, showing that a conscious and strategic use of gaze information is required to disambiguate angry and fearful faces^16^. In addition, the current data implicates a cortical locus for the integration of emotion and gaze information^54–57^. This is consistent with neuroanatomical and neuroimaging studies showing an overlap of cortical systems that process these features. For instance, representations of *conjunctions* of gaze and emotion have been identified in temporal^3,58,59^ and premotor cortices^18^. Neuroimaging and patient studies have also consistently shown the amygdala to be involved in integrating gaze and emotion expression^47,50,53,60,61^. Importantly, it has been suggested that two parallel routes to the amygdala facilitate threat perception^22–25,62,63^. The evolutionary old subcortical route is thought to elicit early responses to unambiguous threat, particularly fearful faces^28^. Alternatively, the cortical route is thought to processes ambiguous threat signals (e.g., fearful faces with direct gaze)^11^. Our data suggest that while a subcortical pathway may facilitate the rapid detection of fearful faces, cortical mechanisms must be involved in linking contextual information that signals their relevance to the observer.

The results from our study failed to show a main effect of gaze direction on suppression times despite previous reports of a direct gaze advantage under b-CFS conditions^35,64–67^. This finding is again consistent with a heightened sensitivity to emotional expression and dedicated brain mechanisms for the rapid detection of fearful and angry faces irrespective of gaze^68^. In support of this, previous research using Garner Interference tasks show that when emotion can be easily recognised, it is processed before gaze interferes^15^. Other CFS studies also suggest the effects of gaze on early face processing are likely to occur after the emotion cue is processed. For instance, studies examining emotional face processing report much shorter mean suppression times (i.e., in the range of 1000–4000 ms)^38,42,69^ compared to studies examining gaze direction (~6000 ms)^35,64–66^. Previous studies reporting a direct gaze advantage using b-CFS have typically used faces with neutral expressions^35,64–67^. Thus, gaze direction might only affect suppression times under CFS when the face expresses a subtle emotion^11,62,63^. In such a case, the positioning of the sclera and iris might be more visible compared to stimuli used in the current study^11^.

Finally, it should be noted that there is currently a debate about whether CFS indeed measures unconscious processing^32–34,49^. In this study, the reported differences in suppression time between fearful and angry faces are assumed to reflect differences in the unconscious processing these stimuli receive while being suppressed. However, it is possible that these differences reflect a bias for fearful faces that manifests at very early post-conscious stages of processing^32,33^. Importantly, however, this interpretation does not detract from our main conclusion that eye gaze direction does not influence emotional face processing in the absence of awareness. Also, it is argued that many previous reports of differences in the preconscious processing of social stimuli may be explained by differences in other stimulus dimensions such as low-level properties like luminance or contrast^30,70–72^. Indeed, it is possible that the main effect of emotion reported in our study was influenced by differences in visibility of sclera between fearful faces compared to angry faces^16^. However, such differences cannot account for our lack of evidence for a modulatory effect of gaze on emotional face processing in the absence of awareness. Specifically, the elegance of our study lies in the design of conjunction stimuli: Threat+ and Threat− categories both comprised fearful *and* angry faces with direct *and* averted gaze. The use of such stimuli allowed us to examine the integration of emotion expression and gaze direction at the earliest stages of face processing while keeping physical low-level properties of the stimuli matched, overall, across conjunction conditions.

## Method

### Ethics statement

All participants provided written and informed consent before participating in this study. They also consented to the sharing of all their de-identified research data on online research repositories (e.g., Open Science Framework). All experimental protocol used in this study – including the sharing of de-identified research data – were approved by the Macquarie University Human Research Ethics Committee (Caruana #5201200021) and were carried out in accordance with the enforced guidelines.

### Participants

Twenty-four healthy subjects (9 females; mean age, 47.13 ± 13.76 years) participated in the study. All participants had normal vision and no psychiatric history or neurological impairment. Participants were either (1) members of the general public who were recruited via online advertisements or our department’s research participation register and were paid for their time, or (2) students from undergraduate psychology units at Macquarie University who received course credit. Our sample size was selected to be comparable to previous CFS studies investigating the effect of gaze direction and emotional expression^70,73^. A power analysis also revealed that the current sample size was approximately double that required to detect a gaze direction by emotion interaction of the same magnitude reported in previous emotion recognition studies using the same stimuli as those used here^18,48,74^.

### CFS stimulus and apparatus

During CFS, a target stimulus (e.g., a face) is presented to an observer’s non-dominant eye. This stimulus is rendered invisible by simultaneously presenting a high-contrast, dynamic mask, to the dominant eye. The mask dominates conscious perception, and ‘suppresses’ perception of the target stimulus, until it ‘breaks’ into conscious awareness. The time taken for observers to detect the stimulus is an index of the target stimulus’ potency in accessing conscious awareness^33,34,75^. In the current study, participants viewed dichoptic displays on a Samsung SynchMaster SA950 HD LED monitor (60 × 34 cm, 120 Hz), through a mirror stereoscope. Participants were seated 70 cm from the screen with their head stabilized in a chin rest. Two red frames (10.4° X 10.4°) were displayed side-by-side on the screen, separated by 21.6^o^ of visual angle. This ensured that the left and right frames were only visible to the left and right eye, respectively. Fusion contours (random noise pixels; width 0.5^o^) were presented within each of the frames to facilitate binocular fusion of the two images presented to each eye. A black fixation point was continuously presented in the centre of each frame (0.2^o^). Participants were asked to maintain fixation on this point for the duration of the experiment. Prior to testing, participants completed the near convergence test to establish eye dominance^76^. Upon setting-up the stereoscope, we ensured that participants perceived a single (i.e., fused) frame when viewing the screen binocularly. We also confirmed that participants could only see one frame when viewing the screen monocularly with each eye.

We used a selection of face stimuli employed by El Zein *et al*.^18^, which were originally taken from the Radboud Faces Database^77^ but adapted such that the subjective intensity of expressed emotions were equated across anger and fear emotion categories. This intensity calibration was informed by subjective ratings obtained from a sample of 19 individuals, and the adaptation was subsequently verified by another sample of 10 naïve individuals (for more extensive details on stimulus adaptation and validation see El Zein *et al*., 2015, p.15–16)^18^. Faces appeared as greyscale images (2^o^ × 3^o^) within an oval to obscure hairlines (see Fig. 1 for example stimuli). The original stimulus set developed by El Zein *et al*., comprised 24 identities varying in emotion intensity across 7 levels (for both fear and anger) across three gaze directions (directed at participant or averted 45° to the left or right). For use in the current study, we selected only the most extreme intensities to elicit reliable discrimination of Threat+ and Threat− based on previous findings from El Zein *et al*.^18^. This resulted in 4 conditions for each identity: 2 emotions (angry versus fearful) * 2 gaze directions (averted versus direct). Importantly, the two Threat+ and two Threat− conditions contained the same two gaze directions and two emotional expressions but differed exclusively in their unique feature conjunctions. Thus, any prioritized preconscious processing of Threat+ stimuli over Threat− stimuli by the visual system (as evidenced by shorter suppression times), could not be explained by the separate features alone. All images were presented against a grey background. In all conditions, face stimuli were presented within the frame visible to the non-dominant eye, whilst high-contrast contour-rich coloured masks (9.2^o^) were presented in the frame visible to the dominant eye to induce interocular suppression and thus render the stimuli temporarily invisible (see Fig. 1. for a detailed description of stimulus development and presentation).

### Procedure

The experiment comprised 384 trials (96 trials per condition), with each stimulus being repeated four times. At the beginning of each trial, both red frames, with fusion contours and the central fixation point were presented against a grey background (see Fig. 1). After 100 milliseconds (ms), the masks were presented within the frame visible to the dominant eye, flashing at a frequency of 10 Hz. At the same time, a face was gradually presented within the frame visible to the non-dominant eye by linearly increasing the stimulus contrast from 0–100% over a one second period. Face stimuli were presented to either the top-left, top-right, bottom-left or bottom-right quadrant of the fusion contour, centred at an eccentricity of 3 degrees of visual angle from fixation. The location of stimulus presentation was counterbalanced such that stimuli from each condition were presented to all quadrants equally. After eight seconds, the contrast of the mask linearly decreased from 100–0% over a two-second period.

Participants were explicitly instructed to report the location of the face stimulus as soon as *any part* of it became visible (i.e., broke suppression) and were not required to make judgments about the face itself. Participants used their right hand only to indicate whether the stimulus was presented left or right of fixation by pressing the left arrow key with their index finger or the right arrow key with their middle finger. Suppression times, the time taken to locate each stimulus, were recorded. Only correct responses were analysed (mean accuracy was above 89% for all conditions: Angry-Direct, *M* = 89.00%, *SD* = 6.07%; Angry-Averted *M* = 90.17%, *SD * = 6.14%; Fear-Direct, *M* = 91.21%, *SD* = 6.14%; Fear-Averted *M* = 91.04%, *SD *= 5.92%). Individual trials in which the participant reported prematurely (<200 ms) or responded after the mask was ramped down were also excluded from the analyses (retained trials: mean = 94.12%, SD = 6.12%). Participants were given a short break half-way through the experiment.

### Analyses

Statistical analyses were conducted using the free software JASP (JASP Team, 2017). The effects of our four conditions on mean suppression times were examined using two-way repeated measures ANOVA.

Suppression times were used as an index of stimulus potency in reaching conscious awareness^33,34,75^. Each individual’s mean suppression times were log-transformed (log_10_) to account for the positive skew of the reaction time data^78,79^. All statistical analyses were conducted on log transformed data, but Fig. 2 summarises untransformed data to facilitate the intuitive interpretation of the results.

## Data Availability

Data used in the reported analyses are available to download from the Open Science Framework: https://osf.io/2dkrq/.
